# Gut microbiome-mediated health effects of fiber and polyphenol-rich dietary interventions

**DOI:** 10.3389/fnut.2025.1647740

**Published:** 2025-08-29

**Authors:** Franziska Meiners, Asiri Ortega-Matienzo, Georg Fuellen, Israel Barrantes

**Affiliations:** ^1^Institut für Biostatistik und Informatik in Medizin und Alternsforschung, Universitätsmedizin Rostock, Rostock, Germany; ^2^Conway Institute of Biomolecular and Biomedical Research, School of Medicine, University College Dublin, Dublin, Ireland

**Keywords:** nutrition, gut microbiome, metabolites, chronic diseases, aging, healthspan, polyphenols, fiber

## Abstract

Dietary components substantially influence aging-related health outcomes through the interaction with the gut microbiome. In this narrative review, we compiled human dietary intervention trials with varying complexities: from simple modifications like the addition of herbs and spices, nuts and beans, to whole-diet patterns such as the calorie-restricted high-polyphenol Green-Mediterranean diet. We show that the addition of fiber- and polyphenol-rich foods consistently enrich short-chain fatty acid (SCFA) producing bacteria such as *Faecalibacterium*, *Eubacterium, Roseburia*, and *Blautia*, and modulate various plasma and fecal metabolites, including increased levels of propionic acid when combining nuts with caloric restriction, increased visceral fat loss mediated by urolithins, and enhanced anti-inflammatory effects, potentially due to synergistic action between SCFAs and polyphenol metabolites. Furthermore, we highlight that relatively simple dietary modifications can produce meaningful microbiome and metabolite shifts, particularly in elderly and metabolically compromised populations, where the microbiome may be more responsive to intervention, and intervention effects are more pronounced. When added to strategies like caloric restriction, these foods can help preserve microbial diversity, maintain beneficial taxa, and enhance anti-inflammatory effects. These insights can inform the development of microbiome-targeted dietary strategies for improving health in high-risk populations.

## Graphical abstract

**Figure:**
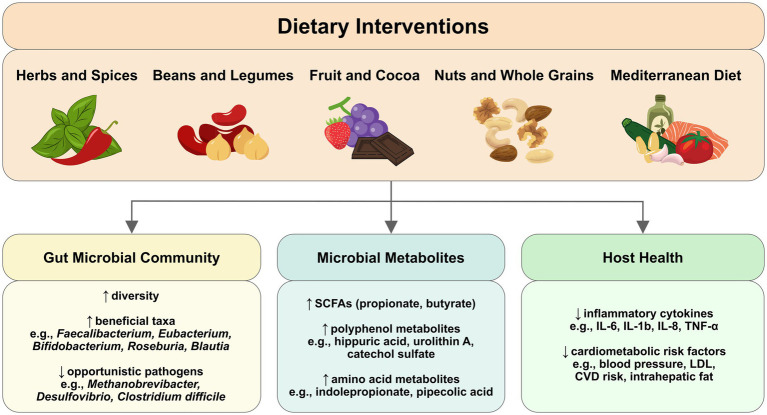
Dietary interventions can modulate gut microbial composition and metabolites that influence host health outcomes. Gut microbiome-targeting interventions can range from simple food additions to more complex dietary patterns, influencing gut microbial composition, diversity, metabolite production, and resulting health benefits. Arrows show the progression from dietary intake through microbial changes to host health outcomes. ↑ indicates increase; ↓ indicates decrease.

## 1 Background: dietary patterns, microbial modulation, and intervention potential

Large-scale, population-based epidemiological studies show that dietary habits strongly influence the risk of disease, mortality, and disability, and the likelihood of healthy aging (1, 2). The quantity and types of nutrients affect metabolic and aging-related pathways (3), and shape the gut microbiome, including microbial diversity, composition, and metabolite production. These microbial changes closely correlate with health status and age-related physiological decline, both reflecting and potentially contributing to deteriorating health (4, 5). Differences in microbial composition and microbially produced metabolites have been consistently identified across a broad range of non-communicable diseases: in cardiovascular disease (6), hypertension (7), metabolic syndrome (8) and diabetes (9), chronic kidney disease (10), colorectal cancer (11), frailty (12), age-related macular degeneration (13) and Alzheimer’s disease (14). This knowledge, together with the widespread prevalence of high-risk dietary habits, i.e., high intake of sodium, meat, sugar, and trans fats, and low consumption of vegetables, whole grains, fruits, nuts, low-fat dairy, and seeds (1, 2) have driven the development of microbiome-targeted dietary interventions.

Such interventions tend to be more effective when they include fiber- and polyphenol-rich foods, which are selectively fermented by gut microbes into health-promoting metabolites such as short-chain fatty acids (SCFAs) and phenolic acids. These metabolites contribute to gut barrier integrity, immune modulation, and metabolic regulation. This fermentation process underlies, at least in part, the health benefits commonly associated with fiber- and polyphenol-rich foods. For example, in patients with Parkinson’s disease, diets high in fiber have been associated with anti-inflammatory SCFA producers and reduced neuroinflammation, while higher sugar intake correlates with potentially pathogenic bacteria (15).

Among health-promoting dietary strategies, interventions enriched in polyphenols, including the addition of specific foods (e.g., spices, legumes, and nuts) as well as whole-diet patterns such as the Mediterranean and Green-Mediterranean diets, can modulate the microbial community and enhance the production of beneficial microbial metabolites (16–18). This review focuses on these dietary interventions, exploring their effects on the gut microbiome, host metabolite profiles and gut barrier integrity, and the mechanisms by which these effects are mediated.

## 2 Microbial metabolism of dietary components

Fermentable polysaccharides, polyphenols, residual peptides and amino acids, and biopolymers that consist of polysaccharides bound to phenolic acids, can be broken down by the colonic microbiota in a series of biochemical reactions, involving different species of microorganisms and intermediary products. The metabolic products from microbial fermentation have metabolic, immunomodulatory, and neurological effects (19), modifying the local gut environment and host health. The composition (and potential benefits) of the metabolic output is determined by availability and composition of substrates present for fermentation, and the functional capacity of the microbiota to break down these components.

### 2.1 Microbial fermentation of dietary fibers and SCFA-mediated host effects

Dietary fibers are complex carbohydrates that escape digestion in the upper gastrointestinal tract and reach the colon, where they serve as substrates for microbial fermentation. Fermentable fibers include a broad range of plant-derived polysaccharides, such as pectin, arabinoxylan, beta-glucans, fructo-oligosaccharides (FOS), galacto-oligosaccharides (GOS), inulin, xyloglucans, and resistant starches (20, 21). These fibers differ in solubility, structure, and fermentability, and have been studied for their influence on gut microbial composition, particularly on fiber-degrading taxa, and the concentration of microbial metabolites (22). While humans lack the enzymes required to break down plant cell wall components, the majority of gut microbes have the functional capacity to do so, and to use them as carbon and energy source (23, 24). The main fermentation end products from fiber are short-chain fatty acids (SCFAs), which have been extensively studied because of their role in metabolic health (25). SCFAs are organic acids containing two to six carbon atoms, with acetate (C2), propionate (C3), and butyrate (C4) being the most abundant, and produced in a ratio of approximately 3:1:1 (26). The key SCFAs, their microbial producers, and host-relevant functions are summarized in Table 1.

**Table 1:** Overview of major short-chain fatty acids (SCFAs) produced through microbial fermentation of dietary fibers in the colon, associated bacterial genera, and reported effects on host metabolism, gut barrier integrity, and immune function.

SCFA	Producing genera	Host-relevant functions	References
Acetate	Produced by the majority of gut bacteria; involved in cross-feeding	Energy source; substrate for butyrate production, stimulates mucin 2 expression, mucus production and secretion	(28, 29, 37)
Propionate	*Akkermansia*, *Bacteroides*, *Dialister*, *Phascolarctobacterium*, *Phocaeicola* (succinate pathway); *Anaerobutyricum*, *Blautia*, *Mediterraneibacter* (propanediol pathway)	Substrate for gluconeogenesis in the liver, Anti-inflammatory, reduces CD4^+^ T cell responses by inhibiting NF-κB and HDAC activity; lowers IL-6, IFN-*γ*, and IL-17 expression	(30, 31, 47)
Butyrate	*Agathobacter*, *Anaerobutyricum*, *Anaerostipes*, *Butyricicoccus*, *Coprococcus*, *Faecalibacterium*, *Gemminger*, *Lachnospira*, *Oscillibacter*, *Roseburia*, *Ruminococcus*	Main energy source for colonocytes; enhances tight junction assembly and wound healing; increases mucin production; inhibits NF-κB; reduces IL-12 and IFN-γ; inhibits HDAC activity and supports anti-inflammatory immune regulation	(19, 31–35, 37, 42–45, 152)

The ability to generate SCFAs is functionally redundant across taxonomically distinct bacteria (27): acetate is formed by the vast majority of gut bacteria while butyrate and propionate are produced by subsets of bacteria that form functionally distinct groups, and multiple products can be generated by the same species (28).

Cross-feeding also takes place; e.g., acetate can increase butyrate production, noted between acetate-producing *Bifidobacterium* and butyrate-producing *Faecalibacterium* (29). Examples of propionate-producing genera are *Akkermansia, Bacteroides, Dialister, Phascolarctobacterium,* and *Phocaeicola* via primarily the succinate pathway, and *Anaerobutyricum* (formerly *Eubacterium halii*), *Blautia* and *Mediterraneibacter* via the propanediol pathway (30, 31).

Butyrate is mostly formed by genera of the highly oxygen-sensitive anaerobic Clostridia families Lachnospiraceae and Ruminococcaceae, and examples include A*gathobacter, Anaerobutyricum, Anaerostipes, Butyricicoccus, Coprococcus, Faecalibacterium, Gemminger, Lachnospira, Oscillibacter, Roseburia* and *Ruminococcus* (31–33).

SCFAs serve as signaling molecules, energy supply, and regulators of metabolism (e.g., insulin sensitivity and fat storage), the immune system, and the gut barrier.

Butyrate provides 70% of the energy requirement of the colonic epithelium (34), facilitates tight junction assembly and promotes wound healing of the intestinal epithelium (35, 36). Butyrate (and acetate) can stimulate mucin 2 (Muc2) expression, mucus production and secretion (37).

SCFAs exert their functions by signaling through surface-expressed (free fatty acid) G-protein coupled receptors on epithelial cells, fat cells, and immune cells (38) or via histone deacetylase (HDAC) inhibition (39). Both signaling routes can regulate T-cell differentiation to induce IL-10-producing regulatory T-cells (40, 41). Butyrate is especially associated with intestinal and immuno-modulatory functions (19, 42, 43), due to its inhibitory effect on NF-κB (44) as well as IL-12 and IFN-*γ* (45), which play a role in chronic low-grade inflammation (46).

Similarly, propionate was shown to be inversely regulated by fasting and refeeding, and to reduce inflammatory CD4^+^ T cell responses by inhibiting NF-κB activity and histone deacetylase activity, leading to lower levels of IL-6, IFN-*γ*, and IL-17 (47).

### 2.2 Protein fermentation and health-relevant microbial metabolites

While our focus is on fiber- and polyphenol-rich dietary interventions, it is important to understand the complete metabolic landscape of the colon, where the availability and type of substrates change along its length, influencing microbial metabolism and the resulting health effects.

In the first part of the large intestine (the proximal colon), microbes primarily ferment carbohydrates, the amount of which gradually decreases toward the descending colon (48), where microbes are specialized to harvest energy from residual peptides and proteins, yielding a more diverse array of metabolic products compared to the fermentation of dietary fibers (23).

Of these metabolites, branched-chain fatty acids (BCFAs) have gained attention because of their association with metabolic imbalances and poor colonic health (49–51). BCFA levels are influenced by diet, and reduced when changing from a Western to a Mediterranean diet, and negatively correlate to butyrate- and acetate generating bacteria, and microbial diversity (52).

Metabolite profiles of prediabetic individuals can feature increased levels of BCFAs (53). *Prevotella copri* and *Bacteroides vulgatus* are two examples of microbes that were found to drive the association between BCFA synthesis and insulin resistance (53). Besides metabolic imbalances, there is also a link between BCFAs and cancer development. A study found that BCFAs produced by *Clostridium symbiosum* led to increased cholesterol synthesis via mTORC1, in turn activating hedgehog signaling, resulting in increased colorectal cancer stemness and tumor growth in mice (54). Other (unfavorable) metabolites from amino acids include the uremic toxin p-cresol, produced from the fermentation of tyrosine, and associated with chronic kidney disease (55), generated by, e.g., *Clostridium difficile* (56).

### 2.3 Fiber-dependent generation of beneficial aromatic amino acid metabolites

Some amino acid metabolites are thought to benefit human health. The effects on colonic and systemic health have been noted for tryptophan metabolite indolepropionate and phenylalanine-metabolite phenylpropionate. Indolepropionate can be produced by members of the *Clostridium* genus or by a two-step transamination and reduction reaction by lactic acid bacteria and *Bifidobacterium* (57). Several *Clostridium* species were found to generate indolepropionate: *C. sporogenes* (58), three strains of *C. cadaveris* (59), the toxin-producing *C. botulinum* (60), as well as *Peptostreptococcus anaerobius CC14N* (59). An important link between the generation of beneficial tryptophan metabolites and dietary fiber intake was established by Qi et al., who found that the intake of fiber-rich foods was most strongly associated with indolepropionate concentrations (50). Interestingly, a study deciphered a mechanism by which specific tryptophan metabolites were generated, which was independent of abundances of tryptophan-metabolizing gut bacteria, but regulated in a substrate-specific manner of metabolic pathways that involved different species and cross-feeding mechanisms (57). In more detail, in an *in vitro* experiment, indole-producing *E. coli* and indolepropionate-producing *C. sporogenes* were shown to compete for tryptophan, and fiber-degrading *Bacteroides thetaiotaomicron* moved the scale in favor of *C. sporogenes*, by cross-feeding monosaccharides to *E. coli*, making more tryptophan available to *C. sporogenes* (57). The results suggest that fermentable fibers can regulate indole production as a beneficial tryptophan metabolite (57), which is in line with studies that found positive associations of indolepropionate with fiber intake (61) but also with polyphenols (62). Finally, it was shown that fiber-mediated regulation of indole generation was not limited to particular species or communities, but is more of a typical phenomenon taking place in the human gut microbiota (57). Indolepropionate concentrations were found to be inversely associated with T2DM risk (50), and lower concentrations were present in patients with heart failure with preserved ejection fraction (HFpEF) in two separate cohorts (63). Indolepropionate is an ligand of aryl hydrocarbon receptors (AhR), and engages with other receptors including pregnane X receptor (PXR) and Toll-like Receptor 4 (63, 64). In a mouse model of HFpEF it was shown that indolepropionate supplementation attenuated diastolic dysfunction, oxidative stress and inflammation by enhancing the nicotinamide adenine dinucleotide salvage pathway, suppression of NNMT (nicotinamide N-methyltransferase) expression, and restoration of nicotinamide, NAD+/NADH, and SIRT3 levels (63). Indolepropionate was also shown to upregulate occludin, which is a tight junction protein (63), thereby strengthening the gut barrier and decreasing intestinal permeability (59). A similar protective action was observed for *Bacteroides fragilis-*derived phenylpropionate in pigs, activating AhR signaling and maintaining the integrity of the intestinal epithelial barrier (65). These examples show how microbial amino acid fermentation can result in metabolites with either beneficial or unfavorable effects on host health, as summarized in Table 2.

**Table 2:** Selected microbial metabolites derived from amino acid fermentation, their substrates, examples of known producers, and host effects as described in the text.

Metabolite	Substrate	Producing/correlated taxa	Reported effects	Health relevance	References
BCFAs	Branched-chain amino acids	*Prevotella copri*, *Bacteroides vulgatus* (correlated taxa); generally negatively associated with SCFA-producers	Associated with metabolic imbalances, insulin resistance and colorectal tumor promotion; linked to low microbial diversity	Unfavorable	(49–54)
p-Cresol	Tyrosine	*Clostridium difficile*	Uremic toxin; associated with chronic kidney disease	Unfavorable	(55, 56).
Indolepropionate	Tryptophan	*C. sporogenes*, *C. cadaveris*, *Peptostreptococcus anaerobius*, *Bifidobacterium*, lactic acid bacteria	Strengthens gut barrier, reduces inflammation and oxidative stress, lowers T2DM and HFpEF risk	Beneficial	(50, 57–60, 63, 64).
Phenylpropionate	Phenylalanine	*Bacteroides fragilis*	Enhances epithelial barrier via AhR signaling (shown in pigs)	Potentially beneficial	(65)

Metabolites include both health-promoting and potentially harmful compounds. HFpEF, heart failure with preserved ejection fraction.

### 2.4 Microbial metabolism of dietary polyphenols

Polyphenols are a diverse group of bioactive plant compounds long recognized for their antioxidative capacity and health benefits (66). Most polyphenols have low bioavailability and require microbial transformation for better bioaccessibility (67–69). There are different classes of polyphenols, such as flavonoids (including flavonols and flavanols), ellagitannins, isoflavones and lignans, differing in chemical structure, complexity and origin, that are converted to phenolic acids by the microbiota (70). Proanthocyanidins are oligomeric flavanols, which are themselves a subclass of flavonoids (71), and are abundant in foods like spices, cocoa, beans, legumes, nuts, berries, grains, tea and fruits (72). Flavonols, on the other hand, are broken down by microbiota-mediated ring-fission, resulting in low molecular weight phenolic catabolites including benzoic acid, phenylacetic acid and phenylpropionic acid, hippuric acid, and valerolactones (70, 72, 73).

Phenolic compounds can modulate gut microbial composition and barrier integrity. For example, mice supplemented with epigallocatechin gallate, a polyphenol found in tea, featured increased *Faecalibacterium, Bifidobacterium* and *Akkermansia* abundances and butyrate production and an anti-inflammatory effect as well as an enhanced gut barrier function in a model of inflammatory bowel disease (IBD) (74).

As described above, fiber played an important role in the generation of indolepropionate from tryptophan. The presence of fiber is often necessary for the beneficial action of polyphenols as well, as described below, but fiber is not a mandatory mediator in this case. For example, a fiber-free diet combined with polyphenols indicated that polyphenol-supplementation can suppress mucus barrier degradation (75). In this experiment, mice fed a high-fat/high-sucrose (HFHS) diet were supplemented with a cranberry-rich extract or water. Akkermansia abundances then reached 30% relative abundance upon the HFHS diet supplemented with the cranberry extract, associated with a number of protective effects, including reduced weight gain and visceral obesity and blunted oxidative stress and inflammation compared to the HFHS control group. A potential explanation for the protective effect of polyphenols was indicated by the significantly higher expression of mucus-encoding Muc2 and tumor suppressing transcription factor Krüppel-like factor 4 (Klf4) in the colon (76) compared to HFHS controls. Significantly higher Muc2 expression points toward the higher production of mucin, thereby maintaining the mucus lining and providing the fermentation substrate for Akkermansia (75).

### 2.5 Health effects of microbially derived polyphenol metabolites

Ellagitannins and their hydrolysis product ellagic acid, found, e.g., in walnuts, berries, pomegranates, pecans and almonds, are known to be metabolized by *Gordonibacter* and *Ellagibacter*, certain *Lachnospiraceae* members and *Enterocloster* species into urolithins (77, 78). *Enterocloster* species harbor a urolithin C dehydroxylase operon, recently identified as a key mechanism for converting urolithin C to urolithin A, potentially explaining inter-individual differences in urolithin A production (78). Urolithin A has attracted particular interest due to its broad health benefits. It has been shown to increase lifespan in model organisms such as *C. elegans* and mice by enhancing mitophagy, improving cellular function, and alleviating systemic inflammation (79, 80). In a mouse model of colitis, urolithin A (derived from caffeic and ellagic acid) was associated with reduced inflammation and improved gut barrier function (81). In humans, urolithin A acts as a direct AhR ligand, repressing the transcription of pro-inflammatory mediators, including IL6 and prostaglandin-endoperoxide synthase 2 (PTGS2) (82–84). Additional studies have reported its role in enhancing mitophagy, improving mitochondrial function and reducing inflammatory responses (85) as well as its association with increased loss of visceral fat (86).

Urolithin A supplementation (1,000 mg) in elderly individuals improved muscle endurance and reduced inflammatory biomarkers while being safe and well tolerated (87).

Polyphenol-rich foods, including strawberries, can enhance urolithin A production and increase abundances of SCFA-producing bacteria. For instance, daily consumption of 500 g strawberries for nine days significantly increased urinary urolithin A and plasma antioxidant capacity (88), and a 10-week dietary intervention based on strawberries increased diversity and Faecalibacterium and Prevotella abundances (16).

Hippurate is another microbially derived metabolite that has been positively associated with gut microbial diversity, polyphenol metabolism, and diets high in fruit and whole grains (89, 90). Elevated hippurate levels have been linked to reduced risk of metabolic syndrome and increased visceral fat loss (86, 89, 91). Additionally, hippurate correlates with higher abundance of *Faecalibacterium prausnitzii* and lower levels of *Ruminococcus* and *Eubacterium*, suggesting a specific role in metabolic health regulation (89). Elevated blood hippurate has also been associated with increased adipose tissue expression of neuroglobin, a neuroprotective oxygen-binding protein primarily expressed in brain neurons (89, 92).

These two microbially derived metabolites exemplify the diverse host benefits of polyphenol metabolism by the gut microbiota.

### 2.6 Microbial breakdown of polyphenol-bound dietary fiber

The microbial fermentation of plant fibers can release bioactive phenolic compounds from the plant matrix. Many polyphenols are associated with dietary fibers through hydrophobic interactions, hydrogen bonding via hydroxyl groups, or covalent ester bonds; they may also accumulate inside vacuoles or become integrated in the plant cell wall (93, 94).

Polyphenol-bound dietary fibers, such as those in whole-grain cereals, have been experimentally shown to have higher antioxidant capacity than polyphenols alone (95). In grains like wheat, corn, rice and rye, the primary structural polysaccharide is arabinoxylan, a pentose sugar polymer, composed of a xylose backbone with arabinose side chains (96, 97). The phenolic compound ferulic acid is ester-linked to these arabinose side chains where it contributes to the structural integrity of the grain cell wall by crosslinking adjacent arabinoxylan polymers (98). The bran, which includes the aleurone and outer grain layers, contains approximately 90% of whole grain phenolic acids, primarily ferulic acid. Most of it is concentrated in the aleurone, which also stores proteins, phytate, and inorganic nutrients (98, 99). Upon reaching the colon, the antioxidant fiber is fermented by gut microbes, for example *Bacteroides* species can cleave the ester bond linking ferulic acid to arabinoxylan, releasing ferulic acid for absorption (100).

### 2.7 Synergy between phenolic acids and SCFAs

Zheng et al., demonstrated that phenolic acid metabolites and SCFAs can act synergistically to reduce inflammation in Caco-2 cells, a commonly used model of the intestinal epithelial barrier (73). The study showed that the combination of butyrate with any of three phenolic metabolites (phenyl acetic acid, benzoic acid, and phenyl propionic acid) at physiologically relevant concentrations significantly reduced IL-8 production more effectively than the individual compounds alone. The anti-inflammatory synergy was mediated through the downregulation of IL-8, TNF-*α*, and VCAM-1, at both gene and protein expression levels (73). The findings suggest that SCFAs and polyphenol metabolites may work synergistically to restore gut homeostasis by reducing inflammation (73).

## 3 Influence of aging and diet on the gut environment

The human gut and its microbiota undergo progressive changes with aging. These include a decline in commensal bacteria, an increase in potentially pathogenic species and greater interindividual variability in microbiota composition. These shifts are accompanied by local changes in the gut environment, including reduced capacity to maintain barrier integrity and an altered inflammatory profile.

### 3.1 Aging of the gut barrier

Studies have shown that impaired barrier function is associated with thinning of the mucus layer, increased intestinal permeability, and, in older individuals, a slower turnover of the intestinal epithelium. These changes are influenced by both dietary factors and the host microbiota (101, 102). The gut barrier plays a critical role in preventing the translocation of microbes, endotoxins and food antigens into systemic circulation, a function essential for avoiding inflammation and sepsis (103, 104).

Fecal microbiota transfer studies highlight the role of the microbiota in barrier function and host aging. When microbiota from aged mice are transferred to young mice, the young mice exhibit increased intestinal permeability along with inflammation in the central nervous system and retina, and altered cytokine signaling (103). Conversely, transferring microbiota from young to old mice can reverse these effects (103).

In humans, intestinal permeability is often assessed using plasma zonulin levels, a physiological modulator of tight junctions secreted by intestinal epithelial cells in response to dietary or microbial stimuli (105, 106). Zonulin lowers the expression of tight junctions and thereby increases intestinal permeability. Elevated zonulin levels have been linked to aging, frailty, chronic obstructive pulmonary disease (107, 108), arthritis (106) and cognitive impairment, particularly during the progression to Alzheimer’s disease (AD) (109). In AD, changes in gut barrier integrity have been suggested as a potential trigger before AD onset (109). Furthermore, microbial encroachment into the inner mucus layer is a feature observed in dysglycemia, independent of obesity (110). Zonulin levels also correlate with metabolic and microbial profiles. Higher zonulin is associated with increased waist circumference, fat mass, and systemic inflammation (111). In contrast, lower zonulin levels are linked to greater microbial diversity, particularly higher abundances of butyrate-producing *Faecalibacterium prausnitzii*, and reduced abundances of *Bacteroidaceae*, *Veillonellaceae*, *Bacteroides*, and *Blautia* (112). These microbial differences are associated with healthier dietary patterns, including greater intake of fiber, omega-3 polyunsaturated fatty acids, vitamins, and minerals (112).

### 3.2 The essential role of dietary fiber in gut homeostasis

Studies in both model organisms and humans have shown dietary fiber is essential for maintaining a homeostatic relationship between host and microbiota.

This becomes especially evident when examining the consequences of fiber deprivation on gut microbiome composition and barrier integrity. In mice, fiber-deprivation leads to significant compositional changes in both the small and large intestinal microbiota (113–115). In the small intestine, a decline in segmented filamentous bacteria has been observed, coinciding with impaired intestinal Th17 and intraepithelial T-cell development and enhanced susceptibility to infection. These alterations were also observed in the offspring, but could only be reversed through fecal microbiota transplantation, not with a high-fiber diet alone (115).

In the large intestine, low-fiber diets are associated with higher abundances of glycan-degrading microbes, particularly *Akkermansia muciniphila*, and intestinal barrier dysfunction (101, 113, 116).

Low-fiber intake has also been linked to increased abundances of IgE-coated commensals, reduced mucus thickness, exacerbated allergic responses and increased inflammation (116). In addition, antitumor immunity is adversely affected by low-fiber intake (117).

Desai et al. demonstrated that both chronic and intermittent fiber-deficiency shifted the microbiota from saccharolytic fermentation to use of host-derived mucus glycans as a fermentation substrate. This shift was characterized by a rapid expansion of glycan-degrading bacteria such as *Akkermansia muciniphila* and *Bacteroides caccae*, alongside a reduction in polysaccharide-fermenting species, without changing plasma levels of propionate or butyrate. The resulting thinning of the mucus layer favored pathogen expansion, increased inflammation and morbidity (113).

Holmberg et al. further showed that fecal microbiota transplantation from individuals on a low-fiber diet failed to induce adequate mucus production in mice. However, increasing fiber intake by 14 g significantly shifted the microbial composition and restored commensal-mucus interaction, mediated by *Blautia* through its production of acetate and propionate (101).

Although *A. munciniphila* is often considered a beneficial genus due to its propionate-generating ability, recent evidence points to a context-dependent role. Derosa et al. found that baseline *A. munciniphila* abundance was a negative predictor of 12-month survival in non-small cell lung cancer patients undergoing immune checkpoint inhibitor therapy (118). Patients with *A. muciniphila* abundances below 4.8% (considered normal) had the longest median survival (27 months), while those with levels above 4.8% had the shortest (8 months). Those patients with no detectable *A. muciniphila* had a median survival of 16 months after treatment (118). Those in the normal range also exhibited higher microbial diversity (Shannon Index) compared to patients with high or absent *A. muciniphila* levels (118). This represents a clear example of a U-shaped association of a microbial biomarker, in which extremes in one direction or the other are associated with the lowest benefits, or even with negative outcomes.

## 4 Dietary interventions: microbiome modulation, microbial diversity, and baseline composition

The microbial response to dietary interventions is partly determined by the initial composition and diversity of the gut microbiota, and the lead-in diet (52). Given the high interindividual variability in microbiomes, responses vary widely. For example, individuals with low abundances of fiber-degrading taxa such as *Prevotella* are more likely to experience an increase in butyrate-producing bacteria (31). In a short-term Mediterranean diet intervention, participants with lower initial diversity exhibited greater variability in microbial response (52). However, even individuals with higher diversity show changes in metabolites profiles that deviated from their initial state, highlighting the importance of considering the metabolome when attempting to define a healthy microbiome and to evaluate intervention outcomes (119).

In the following sections, we will review dietary intervention studies in elderly individuals and those with ongoing disease processes such as prediabetes, obesity, and metabolic syndrome. In these conditions, lower abundances of fiber-degrading bacteria are common (3, 22, 25, 120). We focus on clinical trials involving polyphenol-rich foods, high-fiber and complex-fiber interventions, and whole-diet interventions, organized by increasing complexity: from simple additions of single foods or food groups (sections 4.2–4.6), to food reformulation strategies (section 4.5.3), whole dietary patterns (sections 4.8–4.9), and finally personalized nutrition approaches (section 4.10). We begin with a contrasting example of fiber restriction (section 4.1) to highlight the importance of fiber for maintaining a healthy gut microbiome. This progression allows us to examine how interventions of varying complexity can achieve similar beneficial outcomes through shared mechanisms of increasing SCFA production and polyphenol metabolism. Where relevant, we will refer to previous sections on how dietary components are processed by the microbiome and their influence on host physiology, drawing from observational studies, *in vitro* research, and animal models.

### 4.1 High-protein, low-carbohydrate diets and microbiome effects

Before examining interventions beneficial for health and the microbiome, the following example aims to address the unintended effects on the host and microbiome of a high-protein, low-carbohydrate weight-loss diet. In a clinical trial, 80 overweight or obese postmenopausal women followed an 8-week very-low-calorie diet based on a meal replacement shake (121). The shake contained skimmed milk powder, milk protein, sodium caseinate, maltodextrin, canola and sunflower vegetable oils, artificial sweeteners, and was fortified with minerals, vitamins and inulin. The intervention led to weight loss and transient improvements in glucose regulation. Gut microbiota shifted significantly, with increased abundance of *Akkermansia*, and reduced abundances of plant-metabolizing genera *Roseburia, Ruminococcus, Eubacterium*, which are known to produce SCFAs (see section 2.1). Levels of SCFAs acetate, butyrate and valerate were significantly reduced (121). To explore the role of the microbiome, human fecal samples from trial participants were transferred into mice. This reproduced weight loss but revealed an unexpected enrichment of pathogenic *C. difficile* post-intervention, despite similar baseline abundances pre-intervention. This suggested a diminished capacity of the post-diet to restrict pathogen growth (121). These findings underscore how a complex change in diet composition can lead to multifaceted effects on host biomarkers, involving changes in the microbiome and its metabolic output.

### 4.2 High-polyphenol dietary interventions

#### 4.2.1 The MaPLE trial: substitution of low- with high-polyphenol foods

A randomized controlled trial, the MaPLE trial, evaluated the substitution of low-polyphenol- with high polyphenol-foods in elderly with increased intestinal permeability. Over 8 weeks, participants consumed three daily portions of polyphenol-rich foods, e.g., berries, blood orange, pomegranate juice, green tea, apple and dark chocolate, providing a broad spectrum of polyphenols including proanthocyanidins, tannins and flavonols. The intervention resulted in lower plasma zonulin and fecal calprotectin levels, lower blood pressure, increased abundances of SCFA-producing bacteria, and modulation of multiple cytokines and metabolites (122–124).

Notably, participants with higher zonulin levels were also those with higher BMI and insulin resistance (123). Calprotectin levels (a biomarker used to evaluate gut inflammation) correlated with age, insulin, HOMA index, CRP, IL-6 and TNF-*α* (123, 124). Analysis of the serum metabolome showed that the intervention increased the microbially-produced polyphenol metabolites hippuric acid (see section 2.4), catechol sulfate, 2-methylpyrogallol sulfate and HPPA-S (3-(3-hydroxyphenyl) propanoic acid sulfate) (122). Furthermore, the theophylline metabolites 3-methylxanthine and 7-methylxanthine increased in concentration, while deoxycarnitine, hydroxyhexanoylcarnitine and asparagine were reduced.

Theobromine, a diet-derived metabolite from cocoa, was positively correlated with SCFA-producing genera such as *Roseburia, Butyricicoccus, Faecalibacterium,* and *Lactonifactor,* and negatively with *Methanobrevibacter,* and *Desulfovibrio*. *Faecalibacterium* and members of the *Ruminococcaceae* family increased significantly and inversely correlated with inflammation markers (123). These important findings support that microbially metabolized plant compounds can reinforce gut barrier integrity and reduce systemic inflammation in elderly.

#### 4.2.2 Adding herbs and spices to a Western dish

Another approach to increasing polyphenol intake was tested by adding herbs and spices to a Western-style dish. In a randomized cross-over controlled trial involving obese individuals at risk for cardiovascular disease, this simple dietary addition induced favorable shifts in microbiota composition and host health markers (125). A medium dose (3.3 g) and high dose (6 g) of spice significantly altered community composition (beta-diversity) while the medium dose also increased alpha-diversity (125). Notable increases in *Faecalibacterium*, *Ruminococcus* and the genus *UCG005* from the *Ruminococcaceae* family were observed in the high-dose spice intervention along with reduction in 24-h systolic blood pressure (125, 126).

A secondary analysis found that the medium-dose spice diet significantly reduced fasting plasma IL-6 and postprandial IL-1β, IL-8 and TNF-*α* and modulated monocyte function (127). In another study in healthy Singaporen males, spice intake was associated with higher *Bifidobacterium* abundances and increased levels of the phenolic acids cinnamic acid and phenylacetic acid (see section 2.4) (128).

### 4.3 Interventions with legumes

#### 4.3.1 Bean-based intervention in high-risk obese patients

Colorectal cancer development is strongly influenced by lifestyle factors and adiposity (11, 129, 130). In the BEGONE trial, high-risk obese patients with a history of precancerous colonic polyps or colon cancer participated in a bean-enriched dietary intervention. Participants consumed one cup of beans (providing 16 g dietary fiber and 14 g of protein) alongside their usual diet. This intervention significantly increased the abundances of *Faecalibacterium, Eubacterium,* and *Bifidobacterium,* diversity, and induced broad changes in the host metabolome (131) (Table 3). Metabolic changes included reduced indole, increased levels of in pipecolic acid, the methyl group donor SAM (S-Adenosylmethionine), and trigonelline (131). These metabolites that increased upon the intervention have been associated with anti-inflammatory and potentially anti-aging effects. Pipecolic acid has been shown to ameliorate LPS-induced inflammation in mice and is associated with frequent exercise (132). Trigonelline, a microbial-derived catabolite of niacin and NAD^+^ precursor, is reduced in sarcopenia and is considered a potential therapeutic for age-related muscle decline (133). Furthermore, a reduction in interleukin-10 receptor-*α* and an increase in fibroblast-growth factor-19 (FGF19) were observed. FGF19 controls bile acid and glucose homeostasis, and is associated with increased glucose uptake and energy expenditure (134).

**Table 3:** Intervention effects in the microbiome and the host from trials reviewed in section 4.

Intervention	Inclusion criteria	Microbiome Changes	Host/Metabolite Effects	References
Low-fiber diet shake	Overweight, obesity, menopause	↑ *Akkermansia*, ↓ *Roseburia*, ↓ *Ruminococcus*, ↓ *Eubacterium*	↓ acetate, butyrate and valerate	(121)
Spices	Obesity and CVD risk	↑ *Faecalibacterium*, ↑ *Ruminococcus*, ↑ *Ruminococcaceae_UCG005*, ↑ beta-diversity, ↑ alpha-diversity (medium dose)	↓ IL-6, IL-1β, IL-8, TNF-α	(125–127)
Spices	Healthy	↑ *Bifidobacterium*	↑ cinnamic acid, ↑ phenylacetic acid	(128)
Polyphenol-rich foods	Elderly with increased intestinal permeability	↑ *Faecalibacterium*, ↑ *Roseburia*, ↑ *Butyricicoccus*, ↑ *Ruminococcaceae*, ↑ *Lactonifactor*, ↓ *Methanobrevibacter*, ↓ *Desulfovibrio*	↓ Zonulin, ↓ calprotectin, ↓ blood pressure ↑ catechol sulfate, ↑ Hippuric acid	(123, 124)
Beans	Risk for colorectal cancer, obesity	↑ *Faecalibacterium*, ↑ *Eubacterium*, ↑ *Bifidobacterium*	↑ Pipecolic acid, ↑ SAM, ↑ Trigonelline ↑ FGF19	(131)
Legumes	Prediabetes (45–75y)	↑ *E. rectale*, ↑ *Roseburia hominis*, ↑ *Bifidobacterium*, ↓ *Ruminococcus* spp., ↓ *Bacteroides*	↑ indolepropionate, ↑ aconitic acid, ↑ methylcysteine, ↑ plasma acetate ↓ LDL, ↓ total cholesterol, ↓ HbA1c	(135)
Corn bran or whole grain	Dyslipidemia LDL > 110 mg/dL	↑ *Agathobaculum* (whole grain)	↓ LDL (bran)	(136)
Whole grain	risk for metabolic syndrome	No significant microbiome shift	↑ plasma butyrate ↑ DHPPA-glucuronide, ↑ 2-aminophenol-sulfat ↑ pyrocatechol-sulfate ↑ pyrocatechol-glucuronide ↓ CRP, ↓ IL-6, ↓ IL-1β	(137)
Food reformulation, croissant with seeds and nut-blend	Low habitual intake of fruit and vegetables	No significant microbiome shift	↑ Urolithin A ↓ dipeptidyl peptidase-4	(139)
Nuts, walnuts	Healthy	↑ *Roseburia*, ↑ *Butyricicoccus*, ↑ *Parasutterella*, ↑ *Eubacterium eligens*, ↑ *Lachnospiraceae*, ↑ *Paraprevotella*, ↑ *Lachnospira* ↑ diversity	↑ Urolithin A, ↑ Iso-urolithin A	(142)
Nuts, almonds	Healthy	↑ alpha-diversity ↓ *Bacteroides fragilis*	↓ Ghrelin	(143, 144)
Caloric restriction and nuts	Obese women, cardiometabolic risk	↑ *Ruminococcus, ↑ UCG-002*, ↑ *Ruminococcaceae UCG-005*, ↑ *Blautia*	↑ Propionic acid, ↓ acetate, ↓ CRP, ↓ TNF, ↓ IL-1β, ↓ IL-8	(145, 146)
Med diet – NU-AGE	Elderly across five EU countries	↑ *Faecalibacterium prausnitzii*, ↑ *Roseburia hominis*, ↑ *Eubacterium* spp., ↑ *Anaerostipes*, ↑ *Prevotella copri*, ↑ *B. thetaiotaomicron*	↓ CRP, ↓ IL-17, ↓ secondary bile acids, ↓ p-cresol, ↓ frailty	(147)
Med diet – CORDIOPREV	Coronary heart disease	↑ diversity (MetS only), *↓ Streptococcus*, ↓ *Clostridia*, ↑ *Bacteroidetes*	↓ TG ↓ CVD risk (26–33%)	(8, 148)
Green-Med	Obesity, dyslipidemia	↑ *Lachnospira*, ↑ *Eggerthellaceae*, ↑ *Prevotella*, ↓ *Bifidobacterium*	↑ Hippuric acid, ↑ Urolithin A, ↓ intrahepatic fat, ↓ CHD risk, ↓ methylation age	(17, 86, 149)
Personalized nutrition – PPT	Prediabetes	↑ *Faecalibacterium prausnitzii*, ↑ *Roseburia hominis*, ↑ *Ruthenibacterium lactatiformans*, ↑ *Flavonifractor plautii* ↑ alpha diversity	↓ human cell shedding	(151)

Focus was on SCFA-producing bacteria, diversity, and host effects (specifically, changes in metabolite concentrations). The up and down arrows signify an increase or decrease in the abundance of microbes, or in the concentration of metabolites or cytokines.

#### 4.3.2 Calorie-restricted, legume-enriched diet in prediabetics

A 16-week, calorie-restricted, legume-enriched randomized clinical trial was conducted in 127 Chinese prediabetic individuals, aged 45 to 75 years (135). Participants in the intervention group replaced two daily meals with dishes composed of 100 g of legumes (mixed beans, red kidney beans, or chickpeas), a soy-based meat alternative, vegetables, and a low glycemic index (GI) carbohydrate source (rice or noodles), along with herbs, spices, and vegetable oil. The intervention led to a 40% reduction in total and saturated fat intake, a 33% reduction in salt intake, and an increase in fiber intake of 17 g/day. In addition, participants in the intervention group exhibited significantly greater reductions in LDL cholesterol, total cholesterol, and HbA1c over time compared to controls, indicating improved lipid and glycemic regulation. Each participant provided six stool samples (weeks 0, 2, 4, 8, 12, and 16) for metagenomic sequencing. Taxonomic responses included increases in *Clostridia* (e.g., *Eubacterium rectale, Roseburia faecis, Roseburia hominis*) and *Bifidobacterium*, and decreases in *Ruminococcus* (*R. gnavus, R. torques, R. lactaris*), *Bacteroides* (*B. massiliensis, B. stercoris*), and *Bilophila wadsworthia*. About half the taxa were significantly correlated with dietary fiber intake. Positive correlations were observed for *E. rectale* and *Bifidobacterium*, and negative correlations (and decreasing abundances) for *R. torques, R. lactaris,* and *R. gnavus*. Interestingly, microbial species enrichment and depletion peaked by the second week and remained stable thereafter. Increased plasma metabolites included methylcysteine and pipecolic acid, the organic acid aconitic acid, and indolepropionic acid, which might be produced in higher amounts due to the increased availability of dietary fiber (see section 2.3). Numerous fecal metabolites decreased upon the intervention, including benzoic acid, 2-furoic acid, various carnitines, amino acids, bile acids and fatty acids. Considering SCFAs, only plasma acetic acid was significantly increased, and negatively correlated with HbA1c, LDL-C, TC and TC/HDL-C ratio (135).

### 4.4 Dietary fiber from grains and food reformulation

#### 4.4.1 Corn-based fiber intervention

A 2024 study investigated the effects of different types of corn flour: whole-grain, refined-grain, and a mix of refined grain with corn bran, on lipid markers in 36 adults with LDL cholesterol above 110 mg/dL (136). The combination of refined flour and corn bran, which contained the highest levels of ferulic acid (see Section 2.6), significantly reduced LDL cholesterol over time, with reductions greater than 5% observed in approximately 70% of participants. Meanwhile, the whole-grain flour significantly increased the abundance of butyrate-producing *Agathobaculum* (136), see Section 2.1.

#### 4.4.2 Whole-grain intervention

In contrast, an 8-week whole-grain vs. refined-grain intervention in individuals at risk for metabolic syndrome did not alter community composition, yet it significantly reduced body weight and levels of the inflammatory markers IL-6, CRP and IL-1β (137). In addition, the intervention increased plasma butyrate and urinary excretion of several metabolites, including the alkylresorcinol DHPPA-glucuronide, 2-aminophenol-sulfate (a microbial degradation product of wheat and rye benzoxazinoids), and the phenol metabolites pyrocatechol-sulfate and pyrocatechol-glucuronide (137).

#### 4.4.3 Food reformulation: Fiber-enriched products

Fiber enrichment of convenience foods is a promising strategy to increase fiber intake (138). In a two-week trial, a sourdough-croissant enriched with a diverse fiber blend (from wheat, rye, poppy seeds, walnuts, flax seeds, and others, totaling 4.8 g fiber per 100 g) was compared to a conventional croissant (1.3 g dietary fiber per 100 g) for effects on metabolic variables, the gut microbiome and appetite (139). Exclusion criteria included high physical activity and the habitual intake of fruit and vegetables.

The fiber-enriched croissant increased urinary levels of urolithin A sulfate (see section 2.5), likely due to the addition of walnuts. The intervention also led to reduced levels of dipeptidyl peptidase-4 (DPPIV) and a slight decrease in fasting blood glucose (139). Urolithin A is known to inhibit DPPIV (140), which degrades incretins - hormones that provoke insulin secretion. Inhibition of DPPIV can therefore improve insulin sensitivity (141).

### 4.5 Interventions with nuts

In a recent study involving healthy individuals, the consumption of 23 g of walnuts twice daily increased alpha diversity, and the abundances of 13 genera (Table 3): *Roseburia*, *Rothia*, *Parasutterella*, *Lachnospiraceae* UCG-004, *Butyricicoccus*, *Bilophila*, *Eubacterium eligens*, *Lachnospiraceae* UCG-001, *Gordonibacter*, *Paraprevotella*, *Lachnospira*, *Ruminococcus torques*, and *Sutterella* (142). Notably, the intervention significantly increased urolithin A and iso-urolithin A, which were significantly associated with several taxa including *Gordonibacter*, *Angelakisella*, *Defluviitaleaceae UCG-011*, and *Anaerovoracaceae Family XIII* (142). These findings suggest that polyphenols in walnuts likely contributed to the observed rise in urolithins (see Section 2.5).

In another study with nuts, 57 g almonds consumed daily for 8 weeks increased alpha-diversity and reduced *Bacteroides fragilis* compared to an isocaloric cracker snack (143). A separate energy-restricted study found that a nut-enriched breakfast, compared to a nut-free breakfast, significantly reduced ghrelin levels, suggesting a role in appetite regulation among individuals at risk for cardiometabolic disease (144).

### 4.6 Caloric restriction combined with nuts in overweight women

Two 8-week randomized controlled trials tested the effects of caloric restriction (CR) with or without the addition of nuts in women with high BMI and increased waist circumference and at least one other cardiometabolic risk factor.

In the first trial, participants consumed 30 g cashews and 15 g Brazil nuts daily. Both CR groups (with and without nuts) showed significant changes in beta-diversity, indicating a shift in community composition. However, only the CR-only group exhibited a reduction in alpha-diversity and a significant increase in the Firmicutes-to-Bacteroidetes ratio, changes that were not observed when nuts were added to the diet (145). A reduction in alpha-diversity points to a less stable microbial community.

Fecal SCFA analysis showed that CR and nuts significantly increased fecal propionic acid, and reduced acetate (145). Lactulose-secretion was used to measure intestinal permeability, which did not differ due to treatment, but lactulose secretion was significantly lower in the CR-nut-group compared to CR at the end of the intervention (145). Abundances that differed after the intervention versus baseline included increased *Ruminococcus* and *UCG-002* in the CR-nut group and increased abundances of *Blautia*, and *Ruminococcaceae* genera *UCG-005* and *UCG-*002, and a reduction in *Ruminococcus* in the CR group. Intestinal permeability correlated with fat-loss, IL-8 and *Ruminococcus* abundances (145). These findings suggest that the addition of nuts to caloric restriction may preserve community stability and support beneficial microbial taxa.

A second trial tested the effect of 8 g of Brazil nuts daily combined with CR to CR alone. The CR-nut group showed significantly decreased levels of CRP, TNF, IL-1β and IL-8 indicating an anti-inflammatory effect of the nut-enriched intervention (146).

### 4.7 Mediterranean diet interventions

The NU-AGE randomized controlled trial examined the effects of a one-year Mediterranean (Med) diet intervention in elderly subjects from five countries (Italy, United Kingdom, France, Netherlands, Poland) (147). Increased intake of fibers from vegetables, fruits and whole grains, plant proteins from legumes, polyunsaturated fatty acids from fish and vitamins and reduced fat, alcohol, salt and sugar intake consistently modulated microbiome composition across countries.

Greater adherence to the Med diet was associated with improved cognitive function, reduced frailty and lower inflammation. Key microbial responders included SCFA-producing taxa (see section 2.1) such as *Faecalibacterium prausnitzii*, *Roseburia hominis*, *Eubacterium* species (*E. rectale*, *E. eligens*, *E. xylanophilum*), as well as *Bacteroides thetaiotaomicron*, *Prevotella copri* and *Anaerostipes hadrus*. These taxa were negatively associated with inflammatory markers hsCRP and IL-17 and measures associated with increased frailty; and were further negatively associated with lower production of secondary bile acids and the uremic toxin p-cresol (147). Prior studies have similarly shown reductions in p-cresol following increased fiber intake (55).

The CORDIOPREV trial evaluated the long-term effects of a Med diet versus a low-fat diet in patients with established coronary heart disease over a seven-year follow-up (8, 148). The Med diet was superior in reducing the risk of major cardiovascular events (148). The analysis of fecal samples from a subgroup of 106 male participants revealed distinct baseline microbial compositions among individuals with and without metabolic syndrome. Those with metabolic syndrome had lower abundances of *Bacteroides*, *Prevotella*, *Roseburia*, *Ruminococcus*, and *Faecalibacterium*, and higher abundances of *Streptococcus* and *Clostridia* (8). Interestingly, the intervention only altered microbial composition in those with metabolic syndrome, and not in individuals without metabolic syndrome independent of obesity (8). Most of the compositional differences in participants with metabolic syndrome were reversed after follow-up 2 years later in addition to significantly decreased TG levels in both dietary intervention groups (8). Nutritional intake patterns shifted during the trial: the Med diet group increased their intake of olive oil, nuts and oily fish, and reduced consumption of carbohydrates and saturated fatty acids. The low-fat group increased carbohydrate consumption and reduced fat-intake; both groups reduced their intake of red and processed meats and carbonated drinks. Both groups increased dietary fiber consumption by 2.3 g (Med) and 3.2 g (low-fat) per 1,000 kcal (8, 148).

### 4.8 Calorie-restricted, high-polyphenol Mediterranean diet intervention

The DIRECT-PLUS randomized controlled trial integrated multiple anti-aging strategies and included 294 participants with abdominal obesity and dyslipidemia. Participants were randomized into three groups: healthy dietary guidelines, a Med diet, and a polyphenol-rich, low-red/processed meat Green-Med diet. Green-Med and Med diets were calorie-restricted and included daily consumption of 28 g walnuts. Green-Med participants were guided to consume 3–4 cups of green tea and a 500 mL shake composed of Mankai, an aquatic plant rich in protein and polyphenols, which increased the polyphenol content of the intervention by 800 mg gallic acid equivalents (17). Both interventions led to weight loss, improved cardiometabolic markers, body weight, blood pressure and fasting plasma leptin. Further, the interventions resulted in significant compositional changes: Green-Med increased abundances of *Prevotella, Bacteroides, Lachnospira* and genus *DNF00809* from the *Eggerthellaceae* family and decreased abundances of *Dorea*, *Collinsella* and *Bifidobacterium* (Table 3). Green-Med intervention compositional changes were largely driven by non-core members (17). Interestingly, reduced Bifidobacterium abundances were significantly associated with greater weight loss. In the Med-diet group, Lachnospira, *DNF00809* (*Eggerthellaceae*), *Enterorhabdus*, *Intestimonas* and genus *UCG-003* from the *Erysipelotrichaceae* family significantly increased. Changes in metabolic pathways included a stepwise increase in BCAA (branched-chain amino acid) degrading pathways and a decrease in BCAA biosynthesis pathways, and an association analysis revealed that reduced cysteine biosynthesis was linked to, and mediated, weight loss and reduction in CHD risk (17). Positive effects were specifically attributed to the increased polyphenol content in the Green-med group, including the significantly higher reduction in intrahepatic fat compared to the Med diet (149) and higher circulating levels of hippuric acid, as well as Urolithin A (see sections 2.4 and 2.5) that related to greater visceral-adipose tissue reduction, mediated by increased consumption of walnuts and Mankai (86).

The Green-Med diet also impacted biological age, as assessed by DNA methylation age clocks. At the end of the intervention, a ~ 9-month favorable difference between observed and expected methylation age was observed (150). Greater age attenuation was linked to higher intake of Mankai and green tea, which correlated with elevated urinary metabolite levels of hydroxytyrosol, tyrosol, urolithin A, and urolithin C (150).

### 4.9 Personalized nutrition

A personalized nutrition approach was tested in the post-prandial glucose targeting (PPT) trial in prediabetic individuals. The PPT diet was based on a machine learning algorithm that integrated multiple parameters (meal’s nutrients composition, blood data, anthropometrics, lifestyle and gut microbial features), to predict an individual’s postprandial glycemic response, and then provided recommendations for meals (151). The PPT diet was compared to a Mediterranean diet intervention (participants were encouraged to include whole-wheat bread and grains, legumes, fruits, vegetables, olive oil, fish, poultry and low-fat dairy products in their diet) (151). While both dietary interventions enriched *Faecalibacterium* species, the PPT diet had a more pronounced effect on the gut microbiome and metabolites than the control diet, including increased richness and diversity, and a significant decrease in human cell shedding. Further, the PPT diet increased *Flavonifractor plautii*, *Roseburia hominis*, *Ruthenibacterium lactatiformans* and three strains of *Faecalibacterium prausnitzii* (151) (Table 3).

## 5 Discussion

In this review, we summarized clinical dietary intervention trials conducted in elderly individuals and those at elevated risk for chronic diseases. We focused on fiber- and polyphenol-rich dietary strategies that aim to modulate the gut microbiota to improve host health. Across diverse interventions, we found consistent evidence that even small dietary changes can beneficially shift microbial composition and function, most often reflected in increased abundances of SCFA producers and altered metabolite profiles.

We chose to focus on clinical trials in high-risk populations, where abundances of SCFA producers are generally lower, and intervention effects tend to be more pronounced (51, 121). This is relevant due to several reasons: Western diets typically fall short of recommended fiber intake levels, and our population is aging, intensifying the need to adjust dietary intake to match protective effects against chronic diseases like heart disease and diabetes. Furthermore, reduced microbial resistance to pathogens is often linked to increased intestinal permeability and systemic inflammation, both of which tend to increase with age. This was reflected in the high-protein, low-carbohydrate trial in obese women, where reduced fiber intake led to a bloom of *Akkermansia*, a glycan-degrading bacterium, reduced levels of SCFAs and the emergence of *C. difficile* following fecal microbiota transfer into mice (121). In contrast, the MaPLE trial demonstrated that polyphenol-rich foods can strengthen the intestinal barrier in elderly living in a residential care facility, particularly in those with elevated permeability at baseline. Zonulin and calprotectin levels were reduced, SCFA producers increased, and circulating cytokines were modulated (124). These findings align with the evidence in mice that polyphenols can help preserve gut barrier integrity by suppressing mucus degradation and regulating tight junctions (75).

Simple dietary changes like the addition of herbs and spices can change microbiome composition, diversity and metabolites substantially. Individuals at risk for CVD showed increased abundances of SCFA producers like *Faecalibacterium* and *Ruminococcus,* and reduced blood pressure and inflammatory cytokine levels (125, 126). In healthy individuals, spices led to increased *Bifidobacterium* abundances and cinnamic and phenylacetic acid levels (128). Spices included cinnamon, black pepper, ginger, coriander, parsley and more, suggesting that, besides fibers and “whole” polyphenol-rich foods, polyphenol-rich spices can meaningfully impact the microbial fermentation products and host immune response.

We also reviewed two trials where legumes were the primary intervention food: the BEGONE trial (beans) and the study by Wu et al. (135). Both included obese participants, either with prediabetes or a history of colorectal cancer or polyps, and showed increases in SCFA producers, such as *Faecalibacterium*, *Eubacterium* and *Bifidobacterium*, and increased microbial metabolites such as pipecolic acid and indolepropionate (131, 135). Both of these metabolites have been linked to reduced inflammatory responses and derive from microbial amino acid metabolism, potentially with the involvement of *Bifidobacterium* (57).

While the Mediterranean diet is well established for its cardiometabolic and cognitive benefits (8, 147), similar shifts in microbiome and metabolites can also be achieved with the addition of individual foods like nuts, legumes, and spices, which are often more accessible and easier to implement. These foods contain overlapping classes of fermentable fibers and polyphenols, and frequently result in increased SCFA producers such as *Faecalibacterium*, *Roseburia*, and *Ruminococcus*. These positive microbial responses were seen across multiple trials.

From a public health perspective, food reformulation may be one of the most scalable strategies to improve microbiome-related health outcomes. Trials using fiber-enriched croissants, bran-enriched flours, or whole-grains consistently showed improvements in markers such as LDL cholesterol, fasting glucose, SCFA production, and microbial metabolites like urolithin A (136, 137, 139). These findings support the idea that microbiota-targeted benefits can be achieved through modifying familiar foods, without requiring drastic dietary changes.

The Green-Med diet from the DIRECT-PLUS trial provides an example of a more complex dietary strategy, combining caloric restriction with higher polyphenol intake from walnuts, green tea, and Mankai. Compared to a calorie-restricted Med diet with walnuts, the Green-Med group showed more pronounced changes in microbial composition, SCFA metabolism, and liver fat reduction, as well as a measurable reduction in biological age as assessed by methylation age (17, 149, 150). This points to the potential of multi-component interventions to synergistically target microbiota-mediated health pathways.

A limitation in comparing across trials is the variability in study design, intervention duration, and lead-in diets. Short-term microbial responses are known to be influenced by habitual diet and microbial community diversity (52). In addition, differences in sequencing methods (16S versus metagenomic sequencing) can limit the comparability of microbiota outcomes across studies. Furthermore, in our review, there is a lack of direct head-to-head comparisons between different populations (e.g., young vs. elderly, metabolically healthy vs. compromised) receiving identical interventions. Our conclusion that intervention effects are more pronounced in elderly and metabolically compromised populations is based on synthesis across studies rather than direct comparative trials. These populations typically show lower baseline abundances of beneficial bacteria and compromised barrier function, which may explain their greater responsiveness to dietary interventions. Future studies directly comparing intervention responses across different populations would strengthen these observations.

Additionally, future studies that directly compare complex, multi-component interventions like the Green-Med diet to simpler, more implementable strategies such as replacing low-polyphenol with high-polyphenol foods would be interesting. The goal should not only be to maximize the intervention effect, but also to evaluate real-world applicability. A personalized approach based on baseline microbiota composition or host metabolic profiles may further enhance intervention effectiveness.

Gut microbial dysbiosis becomes more apparent with advancing age and chronic disease. However, the reviewed trials show that the microbiome remains responsive to dietary modulation. Improving dietary patterns by increasing the intake of fiber- and polyphenol-rich foods, such as beans, nuts, legumes, spices, berries, and tea, can help rebalance host-microbiota interactions and reduce modifiable risk factors. These strategies hold promise both for prevention and for targeted metabolic support, particularly in high-risk populations.
